# Effect of Freeze-Thaw on a Midtemperate Soil Bacterial Community and the Correlation Network of Its Members

**DOI:** 10.1155/2018/8412429

**Published:** 2018-06-27

**Authors:** Yinghua Juan, Nan Jiang, Lulu Tian, Xiaodong Chen, Wentao Sun, Lijun Chen

**Affiliations:** ^1^Institute of Plant Nutrition and Environmental Resources, Liaoning Academy of Agricultural Sciences, Shenyang, Liaoning 110161, China; ^2^Institute of Applied Ecology, Chinese Academy of Sciences, Shenyang, Liaoning 110016, China; ^3^Department of Soil and Environment, Shenyang Agricultural University, Shenyang, Liaoning 110866, China

## Abstract

Freeze-thaw (FT) events can influence soil functions. However, the overall impact of FTs on soil bacterial communities, especially in temperate regions, remains unclear. In this study, soil samples were collected from a midtemperate region in the northeast of China, and three incubation tests were then designed with varied FT amplitudes (i.e., at a freezing temperature of −15, −9, and −3°C, respectively), frequencies of FT cycles (i.e., under one, six, and 15 FT cycles, respectively) and soil water content (SWC) values (i.e., at 10 and 30% SWC, respectively). High-throughput sequencing of 16S rRNA gene amplicons was performed and the functional profile was further predicted based on these data, in addition to examinations of bulk microbial properties. Data analyses suggested that, first of all, the FT amplitude significantly influenced the bulk microbial properties and bacterial community (composition and function); certain taxa showed a nonlinear response to the three amplitudes. Next, compared to a single FTC, multiple FT cycles had only minor effects on the bacterial functional capabilities, although the bulk microbial properties changed significantly after multiple FT cycles. In addition, the bacterial response to FTs was influenced by the SWC, characterized by the significantly different bacterial community structures (composition and function) and the opposite trends of enzyme activities. Finally, RDA plots and a correlation network assembled data from all soil samples across the three tests and suggested that bacterial response trajectories changed because some species were influenced mainly by other species (i.e., biotic environment) during FT processes.

## 1. Introduction

In temperate regions, freeze-thaw (FT) events are common during spring and fall as well as mild winters and could significantly influence soil functions, such as microbial responses that play a vital role in soil nutrients [1, 2]. Undoubtedly, FT lyse microbial cells and release their nutrients into soils; on the other hand, the survivals remain active even when soils are frozen [3, 4]. However, studies using distinct soils and methods have always reported inconsistent effects of FT events on microorganisms [3]. For example, in contrast to the decreased microbial biomass detected after FT processes in some systems [5, 6], insignificant effects were also reported on microbial biomass [7, 8]. Likewise, a significant increase in microbial activity was reported in some cases [6, 9], whereas others reported insignificant or significant decrease effects [10, 11]. In addition, FT is mainly dependent on regional climatic conditions and sensitive to climate change [8, 12]. Global climate change is predicted to result not only in an overall warming trend but also in greater variability in precipitation [13], leading to more variable soil temperatures, frequencies of FT cycle (FTC) and soil water content (SWC) values during cold periods [14, 15]. Therefore, the results of many investigations within one study should be compiled to help elucidate the impact of FT on soil microorganisms.

Unlike higher-latitude soils, which harbor a large proportion of microorganisms that are cold-tolerant and cold-adapted, temperate soil microbes are believed to actively adjust to novel environmental conditions caused by FT [11, 15, 16]. In particular, bacteria that survive frost would recover growth more rapidly than fungi when soil is thawing [4, 12]. Previous studies suggested that shifts in bacterial community are essential for their adaptation to soil temperatures [12, 17], and bacterial metabolic profiles would drastically change in temperate soils during thawing periods [11]. Additionally, cooler temperatures induce changes in the community composition of denitrifying-gene-harboring bacteria and the denitrifying functions recover quickly as soon as the soil begins to thaw [12, 18]. However, due to the limited systematic studies within a common soil plot, how bacterial community responds to FT events in temperate soils, especially in the context of climate change, is still unclear.

Here, in addition to examinations of microbial biomass and extracellular enzyme activities, we used high-throughput sequencing to evaluate the effects of three different FT treatments, including* Amplitude*,* Frequency*, and* Soil Water Content (SWC) tests*, on the bacterial communities in a middle temperate soil. Based on the 16S gene sequencing data, the functional capabilities of the bacterial community were also predicted. Through lens of coexistence theory, correlations between species could also influence the bacterial responses to environmental changes [19]. Therefore, we further analyzed the correlations within bacterial community composition and between bulk microbial properties and bacterial community compositional and functional profiles, providing a comprehensive profile of the effects of FT events on soil bacteria.

## 2. Materials and Methods

### 2.1. Experimental Design

The sampling site was located in Gongzhuling City, Jilin Province of China (43° 30′ 23′′ N, 124° 48′ 34′′ E). The area is under a middle temperate semihumid continental monsoon climate and receives an annual average precipitation of 500–650 mm, an annual average temperature of 0.5-0.6°C, and an average frost-free period of 124–140 days. The soil at the study site is classified as black soil (Hapli-Udic Isohumosols in Chinese Soil Taxonomy) [20], with 1.77 g/kg total nitrogen, 29.97 g/kg organic matter, 1.65 g/kg total phosphorus, 13.2% gravimetric (wet) SWC, 24.9% soil water-holding capacity (SWHC, as mass water content), and pH (H_2_O) 5.8.

The topsoil (0–10 cm) samples were collected at the end of October 2013. All samples were homogenized through a 2-mm sieve. Plant debris, large roots, and stones were removed. To measure the soil properties, approximately 50 g of each soil sample was removed and air-dried. The remaining soil samples were stored at 4°C until use.

Three soil FT incubation tests were established in quadruplicate.* Amplitude test*: Fresh soil (the equivalent of 200 g dry soil) was incubated in a PVC chamber at −3, −9, or −15°C for 6 days and then thawed at 2°C for another day, named as 3F, 9F and 15F, respectively. Soil that had been constantly incubated at 2°C was used as a control [3] and labeled as Con.* Frequency test*: Fresh soil (the equivalent of 200 g dry soil) was incubated under conditions similar to those described for the Amplitude test at −3°C for 6 days and then thawed at 2°C for one day; this was considered one FTC and denoted as 1TF. In addition, 6 and 15 FTCs were carried out, labeled 6TF and 15TF, respectively. 1TF, a single FTC, was used as the control [3] in this test. To maintain the same initial conditions, deionized water was added to adjust the SWC to 15% in the* Amplitude* and* Frequency tests*.* SWC test*: The SWC of fresh soil (the equivalent of 200 g dry soil) was adjusted to 10% (air-dried in a 4°C incubator) and 30% (by adding sterile deionized water), and the soil was then homogenized. SWCs of approximately 10% and 30% accounted for 40% and 120% of the SWHCs, respectively. Incubations were carried out under conditions similar to those described above at −3°C for 6 days and then at 2°C for 1 day. For controls, soil samples were constantly incubated at 2°C with the same two SWC levels. 10PF, 10PC, 30PF, and 30PC indicate the soil samples with and without an FT at a 10% and 30% SWCs.

### 2.2. Measurement of Microbial Biomass Nitrogen (MBN) and Extracellular Enzyme Activities

Soil MBN was measured using the chloroform-fumigation extraction method [21]. Protease activity (PA) and nitrate reductase activity (NRA) in the soil were measured according to the method of Kandeler, using potassium nitrate and tyrosine as the substrates, respectively [22].

### 2.3. 16S rRNA Gene Sequencing

Total DNA was extracted using a FastDNA™ Spin Kit for Soil (MP Biomedicals, Solin, OH, USA) according to the manufacturer's instructions. The concentration and quality of the total DNA were determined by a NanoDrop ND 2000 spectrophotometer (Thermo Scientific, USA) and a Qubit 2.0 Fluorometer (Invitrogen, NY, USA). The V4 region of bacterial 16S rRNA gene was amplified using 520F (5′-AYTGGGYDTAAAGNG-3′) and 802R (5′-TACNVGGGTATCTAATCC-3′) primers with an Eppendorf Mastercycler ep gradient thermal cycler (Eppendorf, Hauppauge, NY, USA). Each sample was tagged by an index sequence at the 5′ end of the forward primer for multiplexing to allow simultaneous analyses of several samples in a single sequencing run. PCR amplification was performed in triplicate. Each reaction consisted of a total volume of 25 *μ*L containing 8.75 *μ*L of sterilized water, 5.0 *μ*L of 5× PCR buffer, 5.0 *μ*L of 5× PCR GC-high enhancer, 2.0 *μ*L of dNTP (2.5 mM), 2.0 *μ*L of template DNA (200 ng/*μ*L), 0.25 *μ*L of TaKaRa polymerase (5 U/*μ*L), and 1.0 *μ*L of each primer (10 *μ*M). The PCR were performed as follows: initial denaturation at 98°C for 3 min; 27 cycles at 98°C for 30 s, 50°C for 30 s, and 72°C for 30 s; and a final extension at 72°C for 5 min. Three microliters of each PCR product was examined by agarose gel (2.0%) electrophoresis. Pooled triplicate reactions were purified using the AxyPrep DNA Gel Extraction Kit (Axygen, USA) and qualified through a QuantiFluor™-ST fluorometer (Promega) and an Agilent 2100 Bioanalyzer (Agilent, USA) according to the manufacturer's recommendations. Next, DNA libraries were constructed and evaluated using the Illumina MiSeq platform with a 500-cycle (2X250 paired ends) kit at Personalbio Technology Co., Ltd., Shanghai, China.

The sequences have been submitted to the GenBank database under accession number SRP106450.

### 2.4. Statistical Analysis

Paired-end reads were filtered using a Q20 threshold in Trimmomatic, and reads that were shorter than 150 bp or contained any 5′-barcode sequences as well as reads with primer mismatches were removed [23]. The valid paired-end reads were then merged using FLASH [24]. All of the merged raw sequences were trimmed using the Quantitative Insights Into Microbial Ecology (QIIME) toolkit v.1.7.0 [23]. Sequences that were shorter than 150 bp, contained ambiguous bases, or exhibited a homopolymer longer than 8 bp were removed. Chimeric sequences were identified and removed using UCHIME [25] in mothur (version 1.31.2, https://www.mothur.org/). Operational taxonomic units (OTUs) were clustered with a 97% similarity cutoff using the program UCLUST in QIIME [26], and OTU representatives were searched against the Silva database (Release 119) [27] for taxonomic identification and phylogenetic alignment using the program BLAST in QIIME [23, 28]. The functional capabilities of microbial communities based on 16S datasets and the Kyoto encyclopedia of genes and genomes (KEGG) classification [29] were further predicted using Tax4Fun package in R [30].

Data were normalized to the sample with the lowest number of sequences across all tests. Bacterial *α*-diversity estimated by the Abundance-based Coverage Estimator (ACE) [31] and Faith's phylogenetic diversity (PD) [32] were calculated using mothur. The effects of FT events on bacterial diversity index and bacterial taxa were tested using one-way ANOVA with Least Significant Difference (LSD) test at a* P* <* 0.05* level by SPSS 16.0 (SPSS, Chicago, IL, USA). To compare *β*-diversity, weighted-UniFrac distance matrices were calculated by QIIME. Principal Coordinate Analysis (PCoA), Principal Component Analysis (PCA), and 99% confidence ellipses were used to visualize dissimilarities of the bacterial community structure using the vegan package in R (version 3.2.5, https://www.r-project.org). The dissimilarities between the taxonomic and functional profiles were computed with Mantel test using vegan package in R. Redundancy analysis (RDA) was used to estimate the correlations between bacterial community (composition and function) and bulk microbial properties (including microbial biomass and extracellular enzyme activities) using the vegan package in R. The partial correlation network across the main bacterial OTUs and bulk microbial properties was calculated using a false discovery rate level at 0.05 with the GeneNet package in R and visualized using Cytoscape 3.4.0 [33].

## 3. Results

### 3.1. MBN and the Activities of Extracellular Enzymes

In the* Amplitude test*, the MBN decreased significantly with increasing amplitude, while a significant increase in the PA was only detected in 9F (*P *<* 0.05*, Table 1). The highest and lowest NRA values were observed in 15F and 3F, respectively (*P *< 0.05, Table 1). No significant differences in the MBN were detected between the treatments in the* Amplitude *and* SWC tests*. Surprisingly, in the* Frequency test*, MBN, PA, and NRA increased significantly after more FTCs (i.e., 6TF and 15TF) compared to 1TF (*P *<* 0.05*, Table 1). Moreover, significant differences in either the MBN or PA were only detected between SWCs of 10% and 30% (*P *<* 0.05*) and not between an FT and the control in the* SWC test* (Table 1). NRA was significantly lower at 30% SWC than 10% SWC and significantly increased and decreased after FT at SWCs of 10% and 30%, respectively (*P *<* 0.05*, Table 1).

### 3.2. Bacterial Diversity and Bacterial Community Structure

**Figure 1:**
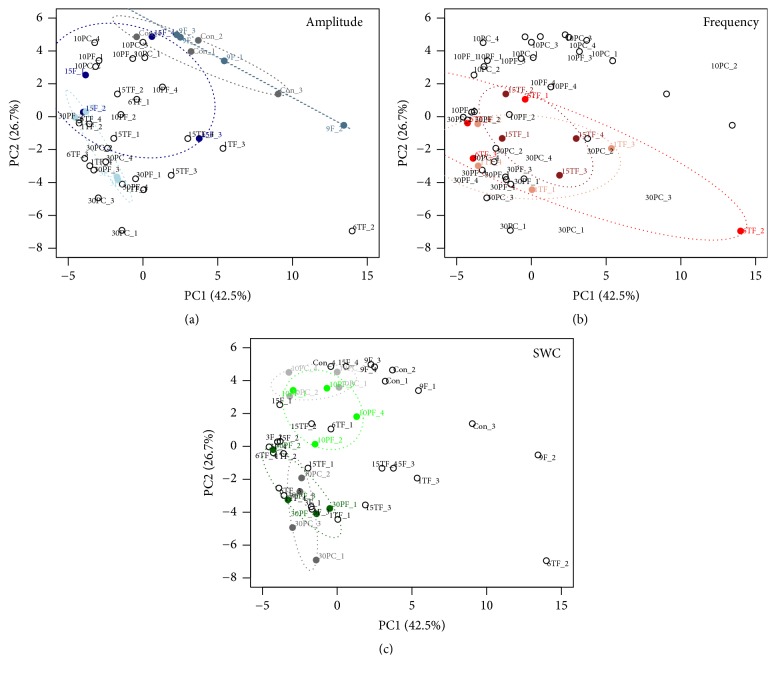
Comparison of the bacterial community structures between different treatments in three tests. Principal coordinate analysis (PCoA) by weighted-UniFrac dissimilarity matrices for the bacterial communities based on OTUs in the* Amplitude test* (a),* Frequency test *(b), and* SWC test* (c). Symbols are colored by different treatments, and ellipses were drawn for each test with a confidence limit of 0.99.

Across all of the tests, a total of 2,525,500 trimmed reads were obtained through the high-throughput sequencing of 16S rRNA gene amplicons, with 25,179-120,294 sequences per sample. The clustering of unique normalized sequences at 97% similarity resulted in 3233–3639 different species-level OTUs per treatment. Significant differences in bacterial *α*-diversity were only detected in the* Amplitude test* (Table 2). Specifically, the ACE and PD values were significantly higher in 3F than in the control (i.e., Con) and the other amplitudes (i.e., 9F and 15F). In addition, phylogenetic *β*-diversity comparisons showed that the bacterial community structures were similar across FTC frequencies (Figure 1(b)), while soil samples could be well separated by different amplitudes and SWC values in the PCoA plots (Figures 1(a) and 1(c)).

### 3.3. Taxonomy Analysis and Functional Prediction

**Figure 2:**
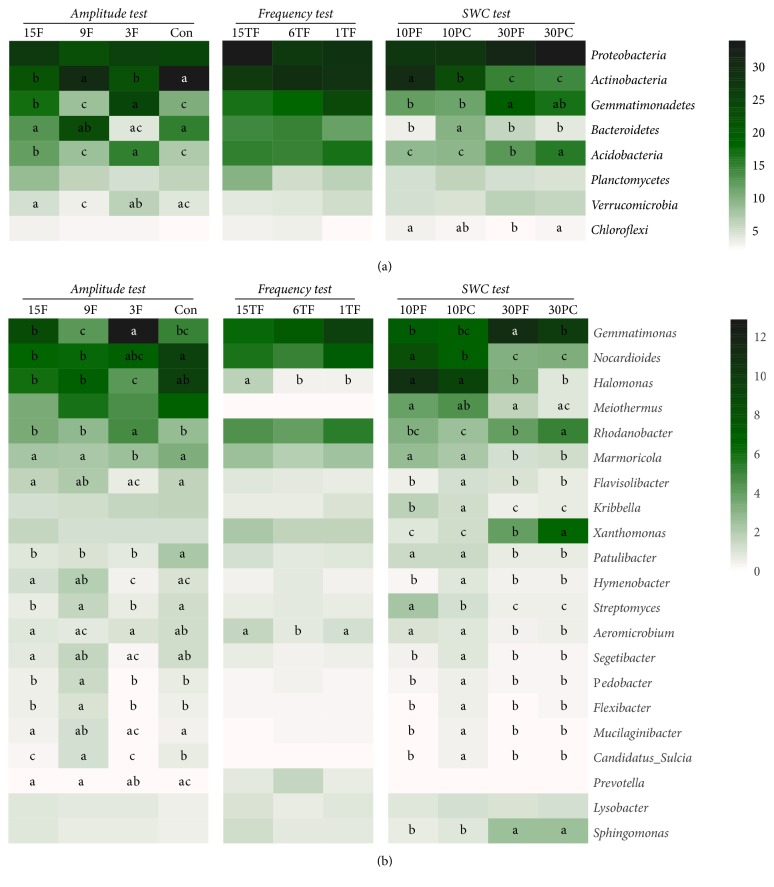
Comparisons of bacterial taxonomy profiles at the phylum (a) and genus (b) levels in the* Amplitude test*,* Frequency test*, and* SWC test*. Only the predominant taxa (above 1% of the relative abundance) in each level are shown. Means of the relative abundance of each taxon for each treatment are compared to the control (*P < 0.05*) and labeled by letters.

The majority of the bacterial sequences in each treatment, ranging from 87.7 to 95.8%, were contributed by the following eight phyla: Actinobacteria, Proteobacteria, Gemmatimonadetes, Bacteroidetes, Acidobacteria, Planctomycetes, Verrucomicrobia, and Chloroflexi (Figure 2(a)). Less than 1% of all sequences on average were unclassified at the phylum level, but more than 40% were unclassified at the genus level.

Comparisons of the predominant taxa for each test are also detailed in Figure 2. In brief, all predominant phyla except for Proteobacteria, Planctomycetes, and Chloroflexi and 16 predominant genera were potentially amplitude-associated, characterized by varied relative abundances in their responses to different FT amplitudes. For instance, compared to the Con, the relative abundance of Gemmatimonadetes and Acidobacteria showed significant increases in 3F and 15F, while the relative abundances of Actinobacteria significantly decreased in 3F and 15F. However, only two dominant genera,* Aeromicrobium* and* Halomonas,* were significantly influenced by FT frequency. In the* SWC test*, after FT, the relative abundance of some taxa significantly changed only at certain SWC values. For example, the relative abundance of Actinobacteria significantly increased after an FT only at 10% SWC, while that of Acidobacteria significantly decreased after an FT only at 30% SWC. Similar situations were also detected at the genera level in the* SWC test*. For example, after an FT, the relative abundance of* Gemmatimonas* significantly increased, and the relative abundance of* Rhodanobacter* and* Xanthomonas* significantly decreased, only at 30% SWC. Likewise, in response to an FT, the relative abundance of* Nocardioides*,* Kribbella*, and* Streptomyces* significantly increased only at 10% SWC.

**Figure 3:**
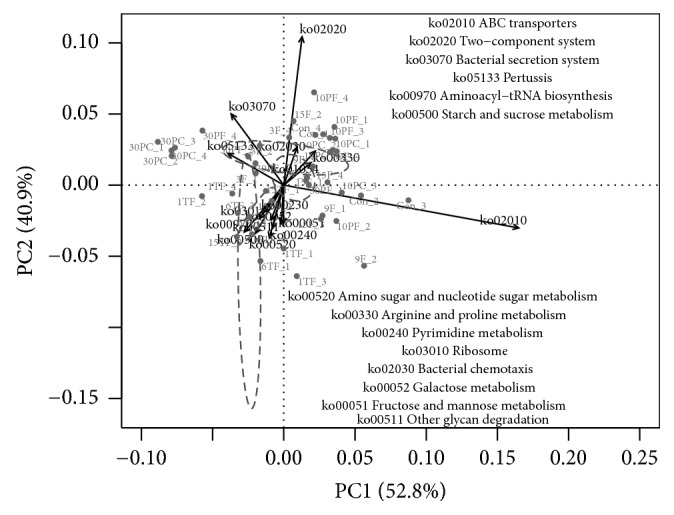
Principal Component Analysis (PCA) of predicting functional profiles (KOs) across all soil samples. KOs with relatively higher vector lengths are arrowed. Ellipses are colored by different conditions (red: amplitudes; dashed line: frequency of freeze-thaw cycle; blue: soil water contents) and drawn for each condition with a confidence limit of 0.99.

Furthermore, Mantel test showed that Tax4Fun-predicted functional profile was significantly correlated with the taxonomic structure across all samples (Mantel statistic r = 0.69,* P *<* 0.001*). PCoA based on the predicting functional profile had a lot in common with the bacterial composition (Fig.  S1). Nonetheless, there were still several subtle differences. In particular, functional capabilities from different soil samples with or without FT could be well separated at each SWC values in the* SWC test*. Likewise, in the* Frequency test*, variations in bacterial functions reduced between replicates of each treatment but increased between treatments. Furthermore, KOs with relatively high vector lengths in PCA plot (Figure 3) belonged to carbohydrate metabolism (ko00500, ko00520, ko00052, and ko00051), nucleotide metabolism (ko00240 and ko00230), membrane transport (ko02010 and ko03070), translation (ko00970 and ko03010), glycan biosynthesis and metabolism (ko00511), amino acid metabolism (ko00330), metabolism of terpenoids and polyketides (ko01054), signal transduction (ko02020), cell motility (ko02030), and infectious disease (ko05133).

### 3.4. Correlations between Bulk Microbial Properties and Bacterial Community (Composition and Function)

**Figure 4:**
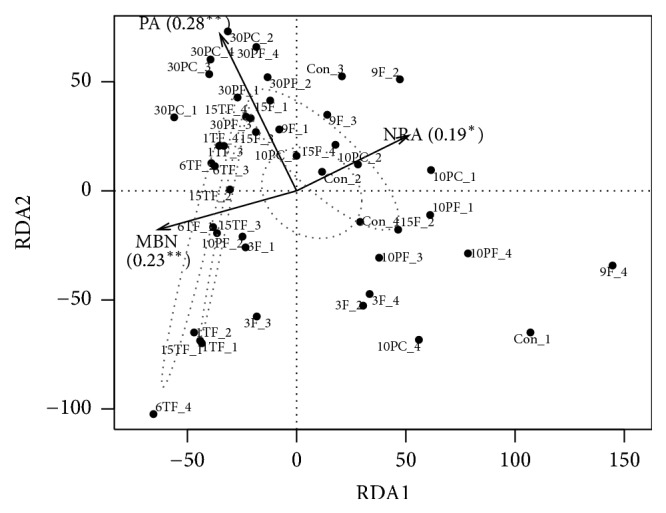
Redundancy analysis (RDA) estimated the relationships between bacterial community composition and bulk microbial properties (including microbial biomass and extracellular enzyme activities) across all soil samples. orthologs are colored by different conditions (red: amplitudes; dashed line: frequency of freeze-thaw cycle; blue: soil water contents) and drawn for each condition with a confidence limit of 0.95.

In the RDA plot, bacterial community composition (>1 % of the relative abundance at the OTUs level) was significantly correlated to MBN, PA (*P < 0.01*), and NRA (*P < 0.05*) across all samples (Figure 4). Specifically, bacterial community compositions from soil samples with different amplitudes were related to both axes, while bacterial community compositions from samples with different frequencies of FTC were mainly related to the first axis, i.e., MBN and NRA. In addition, bacterial community compositions from soil samples at 10% and 30% SWC were mainly correlated to the first (i.e., MBN and NRA) and second (i.e., PA) axes, respectively. Similar results were observed between bacterial functional profile and microbial properties, but only each correlation was relatively stronger (Fig.  S2).

**Figure 5:**
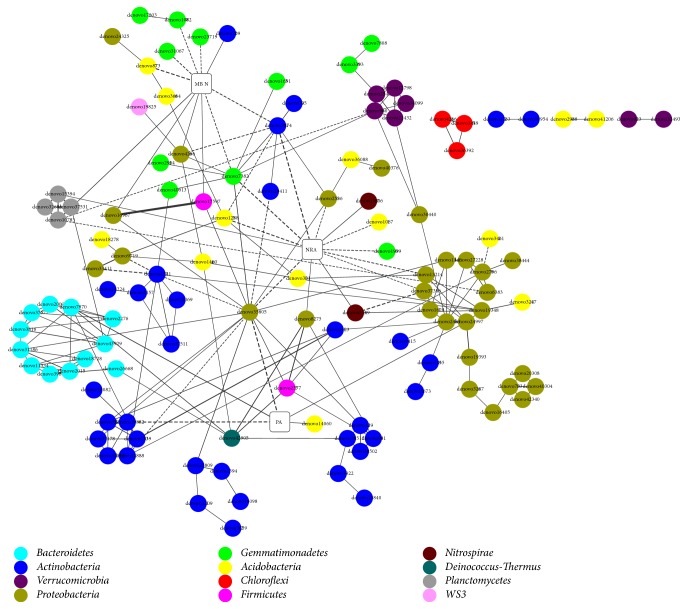
A partial correlation network of the main OTUs (circles, >0.1% of the relative abundance) and bulk microbial properties (squares) across all soil samples. Different colors indicate different phyla. Solid and dashed lines indicate the positive and negative correlations, respectively. PA: protease activity; MBN: microbial biomass nitrogen; NRA: nitrate reductase activity.

A partial correlation network further revealed 206 significant correlations between the main OTUs and bulk microbial properties. In particular, five OTUs belonging to 4 phyla, including Proteobacteria, Bacteroidetes, Actinobacteria, and Acidobacteria, were significantly and directly related to PA (*P < 0.05*, Figure 5). Likewise, a total of 11 and 13 OTUs, two of which were duplicated, showed significantly direct correlations with MBN and NRA, respectively (*P < 0.05*, Figure 5). In addition, most OTUs correlated primarily with others belonging to the same phylum, although some OTUs, such as denova35805, denova42666 (belonging to the phylum Proteobacteria), and denova42905 (belonging to the phylum Deinococcus-Thermus), linked them together (Figure 5).

## 4. Discussion

### 4.1. Nonlinear Effect of FT Amplitude on the Bacterial Communities

Changes in the environment may promote habitat diversification and the establishment of new taxa [34]. In this study, all amplitudes of FT led to significant decreases in microbial biomass, indicating extreme freezing temperature is more detrimental to microorganisms. In addition, variations in the bacterial community structure were significant across different amplitudes. In particular, the relative abundance of the phyla Acidobacteria, Bacteroidetes, Gemmatimonadetes, and Actinobacteria differed the most. Some members of these phyla are bacteria that are active at subzero temperatures, which was previously explored by phylogenetic analysis of ^13^C-labeled 16S rRNA [35]. However, amplitude-associated taxa, with significantly different relative abundance, were not always consistent across the FT amplitudes. For example, among the five amplitude-associated phyla, the relative abundance of Actinobacteria, Gemmatimonadetes, and Acidobacteria changed significantly in 3F and 15F but not in 9F. Likewise, only two of 16 amplitude-associated genera* Nocardioides* and* Streptomyces* showed similar trends across three FT amplitudes. Moreover, in the PCoA plot, the bacterial structures (composition and function) were also more similar between 9F and Con, and the cluster for 3F and 15F was substantially and significantly different than that of Con. These scenarios happen to be similar with results from previous studies in which thawing temperature often changed. For example, frozen cores harbored a significantly different bacterial structure only when thawed at 7°C, in contrast to other temperatures (3°C, 5°C, and 15°C). Alternatively, in a manipulated soil temperature experiment, Deltaproteobacteria, Bacteroidetes, and Gemmatimonadetes showed decreased relative abundances at a 1°C elevated level but returned to their relative abundances in the ambient control at a 2°C elevated level throughout the FT process [36]. Growth of many fungi has often been reported to be nonlinear in response to warming temperature [37], and some bacteria active at subzero temperatures only display activity at certain temperatures [35]. Therefore, the influence of the FT amplitude, in particular of the freezing temperature, on the bacterial community could be significant but nonlinear.

Moreover, our results also showed a substantial increase in PA in 9F. Members of bacteria secrete proteases to depolymerize proteins, producing high quality and quantity of labile substrate [38]. In this case, i.e., soil samples frozen at −9°C, bacterial community could recover rapidly using these labile nutrients during soil thawing. This view also matched the comparatively higher relative abundance of KO orthologs assigned to ABC transporters (ko02010) in 9F. ABC transporters could involve DNA repair besides the uptake of essential nutrients and efflux of toxic molecules [39], suggesting the functional change after an FT with a moderate freezing temperature (i.e., −9°C in our study) exactly coincided with the adaptations necessary for growth at low temperatures based on genomes of psychrophilic bacteria [40, 41]. Meanwhile, high NRA was always in line with a high concentration of the substrate nitrate nitrogen [42], such as that in 9F and 15F here. Nevertheless, PA which has been considered to be critical for soil N mineralization [43] was about 44% lower in 15F than that in 9F, indicating that nitrate nitrogen could mostly be attributed to the death cells rather than N mineralization in 15F. As for 3F, PA and NRA were low, while alpha- and beta- diversity of bacteria changed significantly and substantially, suggesting that reconstruction of bacterial community may happen chiefly. Overall, based on the nonlinear response of bacteria to FT amplitude, extreme (i.e., −15°C) and mild freezing (i.e., −3°C) temperature impact bacterial community more greatly than moderate (i.e., −9°C) freezing temperature, in which higher level of proteolysis and ABC transporters may play an important role.

### 4.2. A small Impact of Multiple FTCs on the Bacterial Communities

The bacterial alpha-diversity (i.e., richness) and beta-diversity (i.e., bacterial community structure) were unexpectedly similar from soil samples after multiple FTCs (6TF and 15TF) and a single FTC. Furthermore, dominant taxa with significantly different relative abundances were not as abundant as those in the* Amplitude* and* SWC* tests, suggesting that multiple FTCs did not have a major impact on the bacterial composition either. Multiple FTCs are always expected to lead to changes in a bacterial community as a result of the combined selective pressures of FT stress and use of liberated substrates [15]. Nevertheless, such unclear directional trends in the general bacterial responses to multiple FTCs have been observed previously [15, 16, 44, 45]: there was a rapid response after the first FTC, similar to that in 3F versus Con in the* Amplitude test* in this study, but the response often diminished after subsequent cycles. The relative unresponsiveness of bacteria to FTCs is often attributed to the fact that the nutrients released by dead microorganisms could maintain the growth of surviving bacteria after an FTC [1, 46]. Since the microbial biomass nitrogen and extracellular enzyme activities, including PA and NRA, increased after multiple FTCs in our study, we proposed that more FTCs may provide sufficient time and substrate for N immobilization and active performance of these surviving bacteria to maintain the community structure. Accordingly, predicting functional profile showed that the relative abundances of KO orthologs assigned to galactose (ko00052), fructose and mannose (ko00051), amino sugar and nucleotide sugar (ko00520), and starch and sucrose (ko00500) metabolisms, all of which affiliated to phosphotransferase system (PTS) used for uptake of carbohydrates, were important to soil samples with multiple FTCs. Soil microbes harbor the potential for carbon metabolism during thaw, especially with a high organic carbon or microbial biomass [41, 47]. The scenarios duplicated if soils underwent multiple FTCs in our study, and our data further suggested that bacteria catalyzes the transporter mainly through the PTS at this moment. Meanwhile, some components of biomolecule synthesis (nucleotide metabolism and translation: ko00230, ko00240, ko003010, and ko00970) also showed increasing trends after multiple FTCs, confirming the active assimilation by bacteria. Besides, the large variation between replicates might slightly override the effect on some of the responses of a bacterial community [15].

### 4.3. Bacterial Responses to FT Differed with SWC Values

Since soil moisture is an important factor affecting bacterial community [48], bulk microbial properties from soil samples with and without FT were compared under two SWC treatments, i.e., at 10% and 30% in the* SWC test*. Drier soil samples (i.e., at 10% SWC) exhibited the lower MBN and PA, indicating that N immobilization and mineralization by bacteria would weaken when water is insufficient. SWC could influence the temperature distribution of soil [3], and dry soils have been shown to freeze more rapidly than wet soils [15]. Therefore, lack of water may enhance the damage to soil microbial cells. In addition, relatively high level of NRA in drier soil samples indicated a high content of nitrate nitrogen, which was accompanied by lower PA and then indirectly confirmed that more microbes might be killed to release nutrients as discussed above. On the other hand, higher SWC was hypothesized to promote the growth and dispersal of microbes [49]. Reasonably, wetter soil samples (i.e., at 30% SWC) harbored a higher MBN and PA in our study. In particular, after FT process, PA was approximately 38% and 76% higher in soil samples at 30% SWC than at 15% (in the* Amplitude test*) and 10% SWC, respectively. Actually, the content of ammonium nitrogen was indeed the highest in soil samples at 30% SWC (data not shown). In general, the increase in SWC could improve the N immobilization and mineralization by microbes in response to FT.

Furthermore, the bacterial community (composition and function) varied considerably at 10% and 30% SWC, suggesting different responses of bacteria between two SWC values. After FT, dominated taxa with significantly different relative abundances were hardly consistent under different SWC treatments. When considering the results from 3F versus Con with 15% SWC in the* Amplitude test* together, for example, the relative abundance of the phylum Actinobacteria increased, decreased, and remained unchanged after an FT cycle at 10%, 15% (in the* Amplitude test*), and 30% SWC, respectively. Moreover, functional profiles suggested that bacteria secrete different functional proteins to survive through bacterial secretion system (ko3070) in wet soils (i.e., at 30% SWC), while ABC transporters (ko02010) accompanied with both chemotaxis (ko02030) and two-component signal transduction systems (ko02020) should dominate the nutrient acquisition in dry soils (i.e., at 10% SWC). In other words, bacterial sense, response, and adaptation to FT may be more complicated when soils are dry. A beta-diversity comparison also suggested that the bacterial community structure of soil samples that experienced an FT cycle changed less at 10% and 30% SWC, suggesting that a sudden extreme of SWC may limit the bacterial reconstruction in response to FT. Moreover, the bacterial community structure of soil samples constantly incubated at 2°C at 30% SWC was more similar to that of soil samples experiencing an FT cycle at 15% SWC, indicating that higher SWC, simulating an increasing in precipitation, may cause a similar effect on the bacterial community to FT with a mild freezing temperature.

### 4.4. Changes in Bacterial Response Trajectories during FT Events

RDA plots assembled data from all soil samples across the three tests. Results showed that N immobilization (MBN), mineralization (PA), and denitrification (NRA) were significantly related to the bacterial community (composition and function) with different FT amplitudes, while N immobilization (MBN) and denitrification (NRA) were mainly correlated with bacterial community (composition and function) with different frequencies of FTC. Additionally, compared with soil samples at 15% SWC which is close to the natural condition, the bacterial community composition at higher SWC (i.e., 30% SWC) was mainly driven by higher N mineralization (PA), but the bacterial community composition at lower SWC (i.e., 10% SWC) was mainly related to lower N immobilization (MBN) and higher denitrification (NRA). Changes in response trajectories under different filter conditions may contribute to the divergent responses of bacteria [36]. In this view, species of community pass through both environmental (abiotic) and biotic filters based on coexistence theory [19].

In our study, the correlation network showed that most bacterial species always coexisted positively with others belonging to the same phyla, such as Bacteroidetes, Verrucomicrobia, Planctomycetes, Chloroflexi, and Proteobacteria in particular. Bacterial groups that tend to coexist could indicate potentially cooperative activities [19, 50], just like the correlations within these phyla during FT events. We proposed that the majority of these phyla could be influenced by the biotic environment, especially within the same phyla. Moreover, species of Actinobacteria were broadly divided into five groups with relatively less correlations within the phylum but more correlations with OTUs from other phyla. Actinobacteria can maintain their metabolic activities under low temperatures [51] and thereby probably cross-feeding or competing with other species based on their positive or negative correlations. Likewise, some members of Proteobacteria were also essential to link other groups. Denovo35805, for example, significantly correlated with 14 OTUs belonging to five phyla. The OTUs belonging to the genus* Ochrobactrum* are known to convert soil substances [52], thereby playing an important role in bacterial response to FT events. Meanwhile, Denovo35805 also significantly and negatively correlated with PA and NRA, implying that this OTU may be driven by abiotic environment and then influence other species as a biotic filter. On the contrary, most OTUs of the phylum Acidobacteria were always significantly related to OTUs from the phylum Proteobacteria, indicating that members of Acidobacteria were filtered mainly by biotic environment during FT processes. The survival of oligotrophic Acidobacteria from FT events ought to be dependent on other species, especially on some members of Proteobacteria. In addition, the majority of OTUs belonging to Gemmatimonadetes in the network, approximately 55%, showed significantly negative relationships with MBN or NRA rather than other bacterial species. Gemmatimonadetes had been proposed to be specifically adapted to dry soils and may be outcompeted when soil water content is available [53, 54], and FT is a similar phenomenon as drying-rewetting [4]. Therefore, it is reasonable that Gemmatimonadetes species should be filtered mainly by abiotic environment during FT processes. Overall, in addition to some certain taxa that changed significantly, the community assembly is also a vital element, which could influence the bacterial response under different FT conditions, and needs to be paid more attention in future.

## 5. Conclusions

In this study, three microcosm FT incubation tests of a midtemperate soil were designed in the context of climate change. Unexpectedly, multiple FTCs showed little influence on the bacterial community composition. Combined with the higher microbial biomass, enzyme activities, and stronger carbohydrate metabolism involving PTS after multiple FTCs, the sufficient time and substrate for N immobilization and active performance of these surviving bacteria could well explain the stable community composition. In addition, FT amplitude and SWC could substantially influence the bacterial community compositional and functional profiles, besides the microbial biomass and enzyme activities. Specifically, the bacterial community changed nonlinearly in response to different FT amplitudes, and the response of the bacterial community in drier soils was quite a contrast to that in wetter soils. Accordingly, the survivals should acquire nutrients through different transporters at varied FT amplitudes or SWC values. We supposed that the bacterial response trajectories changed due to the effects of not only abiotic (environmental) but also biotic (other bacterial species) filters. Overall, a comprehensive profile of the effects of FT events in the content of climate change on soil bacteria was provided for further investigations.

## Figures and Tables

**Table 1:** Soil MBN (mg N kg^−1^ dry soil), PA (mg tyrosine kg^−1^ dry soil h^−1^), and NRA (*µ*g NO_2_^−^-N/g dry soil day^−1^) for all of the treatments.

	Amplitude test				Frequency test			SWC test			
	15F	9F	3F	Con	15TF	6TF	1TF	10PF	10PC	30PF	30PC
MBN	1.89 (0.17) c	2.18 (0.12) c	2.77 (0.07) b	3.50 (0.85) a	4.85 (0.24) b	5.41 (0.21) a	2.79 (0.02) c	2.32 (0.14) b	2.28 (0.02) b	2.86 (0.26) a	3.10 (0.10) a
PA	115.9 (4.9) b	205.7 (6.6) a	130.4 (8) b	120.8 (3.4) b	154.2 (2.6) a	132.1 (2.7) b	118.0 (0.5) c	102.5 (4.8) c	100.7 (3.6) c	179.9 (1.1) b	221.2 (6.9) a
NRA	1.33 (0.00) a	1.22 (0.01) b	0.95 (0.01) c	1.17 (0.03) b	1.05 (0.01) b	1.19 (0.03) a	0.95 (0.01) c	1.32 (0.02) a	1.24 (0.02) b	1.08 (0.05) d	1.19 (0.00) c

Values are given as the mean (SD), n=4.

MBN, PA, and NRA indicate microbial biomass nitrogen, protease activity, and nitrate reductase activity, respectively.

Different letters indicate significant differences among the treatments in each test (Tukey's HSD test, n=4) at* P < 0.05*.

**Table 2:** Indices estimating the bacterial alpha-diversity of each treatment.

	Frequency test			Amplitude test				SWC test			
	15TF	6TF	1TF	15F	9F	3F	Con	10PF	10PC	30PF	30PC
ACE	3282 (251) a	3264 (274) a	3172 (159) abc	3082 (259) abc	2961 (221) bc	3343 (144) a	2910 (257) c	3114 (138) abc	3205 (174) ab	3204 (36) ab	3185 (143) abc
PD	251 (18) a	249 (20) a	238 (11) ab	206 (21) d	217 (19) bcd	231 (13) abc	208 (17) d	216 (12) cd	226 (13) bcd	211 (3) cd	213 (10) cd

Values are given as the mean (SD), n=4.

ACE and PD indicate Abundance-based Coverage Estimator and Faith's phylogenetic alpha-diversity, respectively.

Different letters indicate significant differences among all the treatments (Tukey's HSD test, n=4) at *P* < *0.05*.

## Data Availability

The data used to support the findings of this study are available from the corresponding author upon request.

## Supplementary Materials

Supplementary Materials Supplementary figures include the comparison of the bacterial functional profiles between different treatments in three tests and the relationships between bacterial functional profiles and bulk microbial properties across all samples using PCoA and RDA, respectively.
