# Supplementary material for: Effect of Freeze-Thaw on a Midtemperate Soil Bacterial Community and the Correlation Network of Its Members
Source: Biomed Res Int. 2018 Jun 27;2018:8412429. doi: 10.1155/2018/8412429 (PMC6040300; doi:10.1155/2018/8412429)
Supplement: Supplementary Materials — Supplementary figures include the comparison of the bacterial functional profiles between different treatments in three tests and the relationships between bacterial functional profiles and bulk microbial properties across all samples using PCoA and RDA, respectively. [file 8412429.f1.pdf]

Effect of freeze-thaw on a mid-temperate soil bacterial community and the correlation network of its members

Nan Jiang[2#], Yinghua Juan[1#], Lulu Tian[3], Xiaodong Chen[2], Wentao Sun[1], and Lijun Chen[2*].

Running title: FTs affect soil bacterial community

[1]Institute of Plant Nutrition and Environmental Resources, Liaoning Academy of Agricultural Sciences, Shenyang 110161, Liaoning, China; [2]Institute of Applied Ecology, Chinese Academy of Sciences, Shenyang 110016, Liaoning, China; [3]Department of Soil and Environment, Shenyang Agricultural University, Shenyang 110866, Liaoning, China

[*]**Correspondence:**

Lijun Chen

ljchenchina@hotmail.com; ljchen@iae.ac.cn

Wentao Sun

wentaosw@163.com

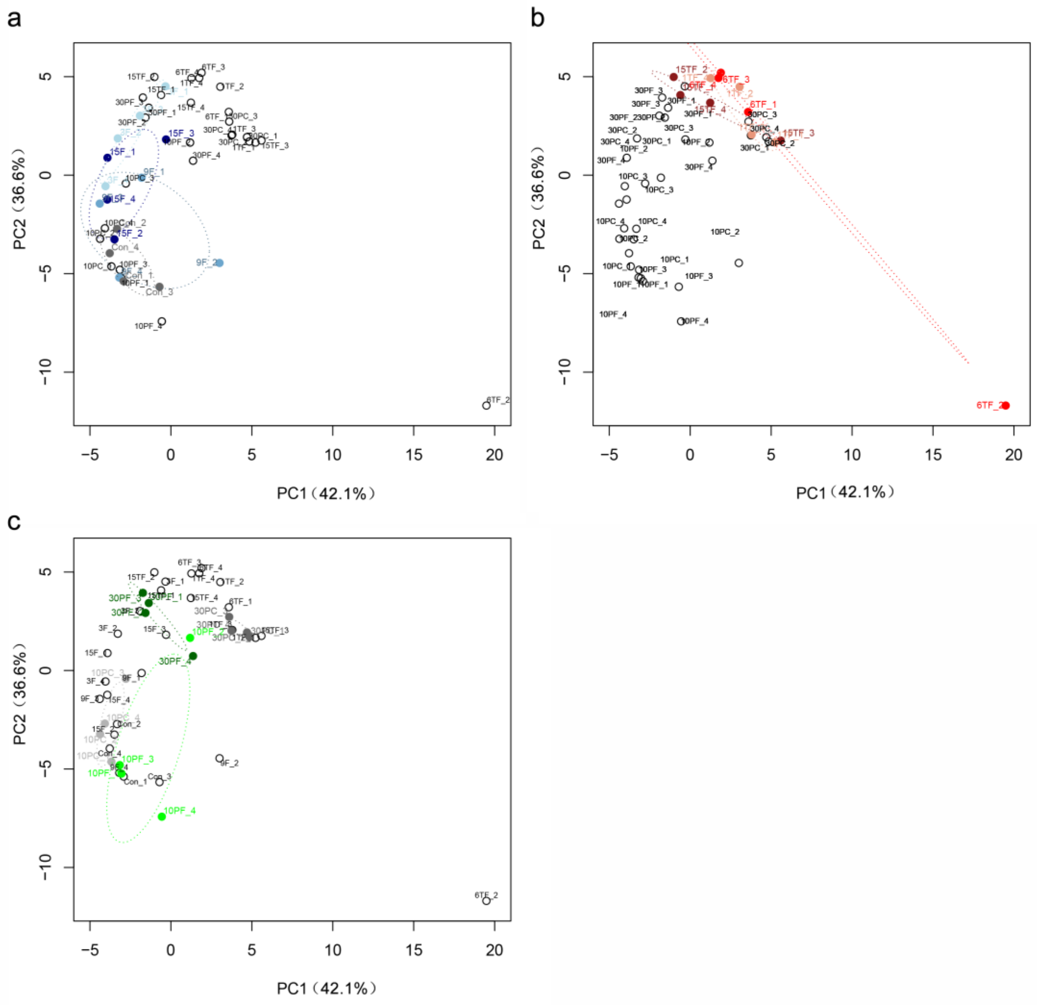

Fig. S1 Comparison of the bacterial functional profiles between different treatments in three tests. Principal coordinate analysis (PCoA) by Bray–Curtis distance matrices for the bacterial KO orthologs based on the Kyoto encyclopedia of genes and genomes (KEGG) classification in the *Amplitude test* (a), *Frequency test* (b), and *SWC test* (c). Symbols are colored by different treatments, and ellipses were drawn for each test with a confidence limit of 0.99.

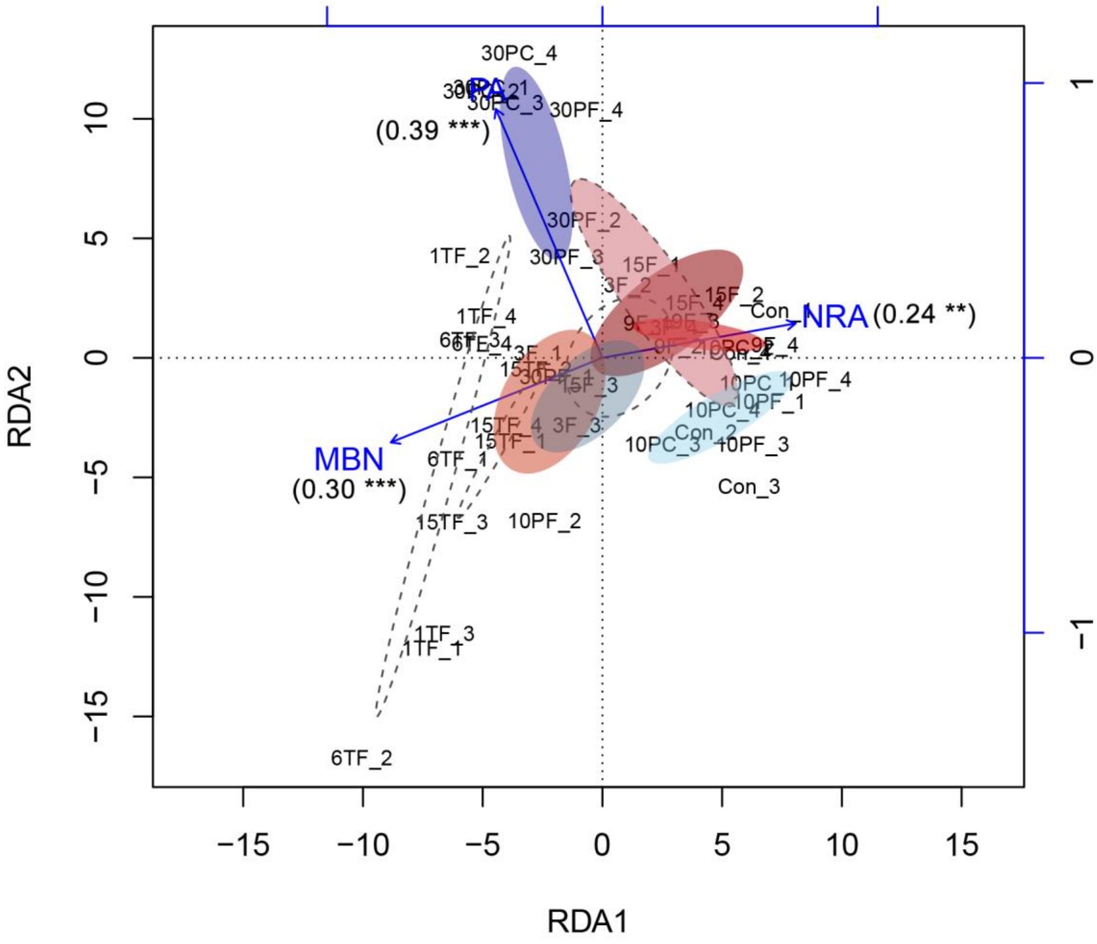

Fig. S2 Redundancy analysis (RDA) estimated the relationships between bacterial functional profiles (i.e., KO orthologs based on the Kyoto encyclopedia of genes and genomes (KEGG) classification) and bulk microbial properties (including microbial biomass and extracellular enzyme activities) across all soil samples. Ellipses are colored by different conditions (red: Amplitudes; dashed line: Frequency of freeze-thaw cycle; blue: soil water contents) and drawn for each condition with a confidence limit of 0.95.
